# Inhibition of Sophocarpine on Poly I: C/D-GalN-Induced Immunological Liver Injury in Mice

**DOI:** 10.3389/fphar.2016.00256

**Published:** 2016-08-12

**Authors:** Yin-Qiu Huang, Peng-Yan Li, Jia-Bo Wang, Hou-Qin Zhou, Zhi-Rui Yang, Rui-Chuang Yang, Zhao-Fang Bai, Li-Fu Wang, Jian-Yu Li, Hong-Hong Liu, Yan-Ling Zhao, Xiao-He Xiao

**Affiliations:** ^1^Pharmacy College, Chengdu University of Traditional Chinese MedicineChengdu, China; ^2^Department of Pharmacy, 302 Military Hospital of ChinaBeijing, China; ^3^China Military Institute of Chinese Medicine, 302 Military Hospital of ChinaBeijing, China; ^4^Research Center for Clinical and Translational Medicine, 302 Hospital of People’s Liberation ArmyBeijing, China; ^5^Department of Integrative Medical Center, 302 Hospital of People’s Liberation ArmyBeijing, China

**Keywords:** NK cell activity, Sophocarpine, NKG2D-DAP12, immunological liver injury, Poly I: C/D-GalN

## Abstract

Increasing evidence has suggested that natural killer (NK) cells contribute to the pathogenesis of human immunological liver injury (ILI). Previous studies have demonstrated that Sophocarpine exerts activity in immune modulation. It also has a therapeutic effect on liver protection in that it can alleviate liver fibrosis by suppressing both the activation of hepatic stellate cells and the proliferation of the activated hepatic stellate cells. However, whether Sophocarpine protects the liver by regulating NK cell activity remains unclear. In this study, the modulating effect of Sophocarpine on NK cells in the liver was investigated. The results showed that Sophocarpine dramatically decreased the production of pro-inflammatory cytokines and attenuated the liver injury induced by Poly I: C/D-GalN in C57BL/6- mice. More importantly, Sophocarpine pre-treatment significantly suppressed NK cell activation and downregulated the expression of NKG2D, a receptor responsible for NK cell activation. Moreover, the protein levels of DAP12, ZAP76 and Syk decreased, as did their corresponding mRNA levels. Overall, our study demonstrates that Sophocarpine inhibits NK cell activity, thus making it a promising therapy for ILI.

## Introduction

Immunological liver injury is a common type of liver damage that is characterized by the severe infiltration of a large number of inflammatory cells into the liver tissue, which leads to an inflammatory response and then to immune response-based liver damage (Hajime and Gregory, 2003; Nakamoto and Kaneko, 2003; Tsutsui et al., 2003; Yin and Ding, 2003). As a predominant organ responsible for innate immunity, the liver plays a critical role in the maintenance of homeostasis, and ILI has attracted increasing concern. NK cells, which are an essential part of the innate immune system, exert a quick response when the human body is invaded by exogenous pathogens or by virus-infected and transformed cells (Trinchieri, 1989; Scott and Trinchieri, 1995; Biron et al., 1999). However, it has been shown that NK cells can kill hepatocytes and cause liver damage when they are excessively activated due to stimulation by viral infection or chemical agents. In this circumstance, their toxicity also correspondingly increases, which results in ILI (Dong et al., 2004). The activity of NK cells is regulated by a balance of positively and negatively transduced signals that are initialized from cell surface receptors (Raulet, 2004; Lanier, 2005). NKG2D, a receptor on the NK cell membrane, has been the most extensively studied for its essential role in NK cell activation. NKG2D is a lectin-like type 2 transmembrane protein and irritant immune receptor that typically couples with signaling adaptors that contain an immune-receptor tyrosine-based activation motif via interactions specified by their transmembrane regions. Furthermore, NKG2D can associate with multiple adaptors, include DAP12. Reportedly, the elimination of the DAP12 signaling complex can block the activating responses initialized by NKG2D (Lanier, 2005). In this study, NKG2D and its associated adaptor DAP12 are targeted.

**FIGURE 1:**
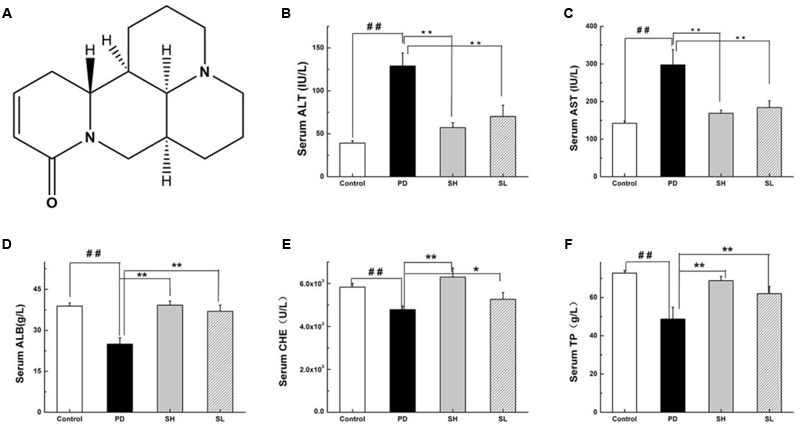
**Effects of Sophocarpine on serum in the liver of Poly I: C and D-GalN-induced mice.** Mice were treated with 7.5 μg/20 g Poly I:C and 25 mg/20 g D-GalN, and they were pre-treated with different doses of Sophocarpine. **(A)** The structure of Sophocarpine. The figure shows the five liver function markers in the serum: **(B)** ALT, **(C)** AST, **(D)** ALB, **(E)** CHE, and **(F)** TP. *N* = 20. Data are expressed as the mean ± SD. ^##^*P* < 0.01 compared with control group; ^∗∗^*P* < 0.01, ^∗^*P* < 0.05 compared with PD group. Poly I:C and D-GalN group (PD group), high-dose Sophocarpine group (SH group) and low-dose Sophocarpine group (SL group).

Sophocarpine (**Figure 1A**) is a quinolizidine alkaloid extracted from *Radix Sophorae Subprostratae*, a traditional Chinese herb that has been used in the clinic for nearly 2000 years in China. Similar in structure to matrine and oxymatrine, Sophocarpine has two quinolizidine cycles in its basic nucleus. It has been reported that Sophocarpine exerts an activity in immune modulation (Zhang et al., 2007). It has also been found that Sophocarpine is an effective agent in the treatment of viral myocarditis for both its antiviral and antiarrhythmic properties (Zhang et al., 2006). Furthermore, Sophocarpine has a therapeutic effect on liver protection in that it can alleviate liver fibrosis by suppressing both the activation of hepatic stellate cells and the proliferation of the activated hepatic stellate cells (Yang et al., 2011). In spite of those experimental studies and case reports, little work has mentioned the effect of Sophocarpine on NK cell activity. Further, whether Sophocarpine can alleviate ILI induced by the excessive NK cell activation is also unclear.

The aim of this study is to fully explore the effects of Sophocarpine on the prevention of Poly I: C/D-GalN-induced NK cell over-activation in the liver and consequent ILI. It also clarifies whether Sophocarpine may suppress the excessive activation of NK cell by downregulating NKG2D and its adaptor DAP12. According to our research, the results indicate a prospective use of Sophocarpine in the therapy of ILI, and our study provides a meaningful reference for ILI treatment in clinic.

## Results

### Effects of Sophocarpine on Serum in the Liver of Poly I: C and D-GalN-Induced Mice

First, we investigated whether Sophocarpine attenuates Poly I: C/D-GalN-induced ILI. The serum levels of ALT and AST, two conventional indices indicating liver injury, were measured. Compared with the control group, the ALT and AST in the Poly I: C/D-GalN group were significantly increased by Poly I: C/D-GalN (**Figures 1B,C**). The administration of high-dose (SH, 120 mg/kg) and low-dose (SL, 60 mg/kg) Sophocarpine significantly reduced the serum levels of ALT and AST, respectively, compared with the PD group. Meanwhile, Poly I: C/D-GalN treatment decreased the CHE, ALB and TP levels in the serum, although this effect was attenuated by Sophocarpine (**Figures 1D–F**).

### Attenuation of Sophocarpine on Immunological Liver Injury in Mice

**FIGURE 2:**
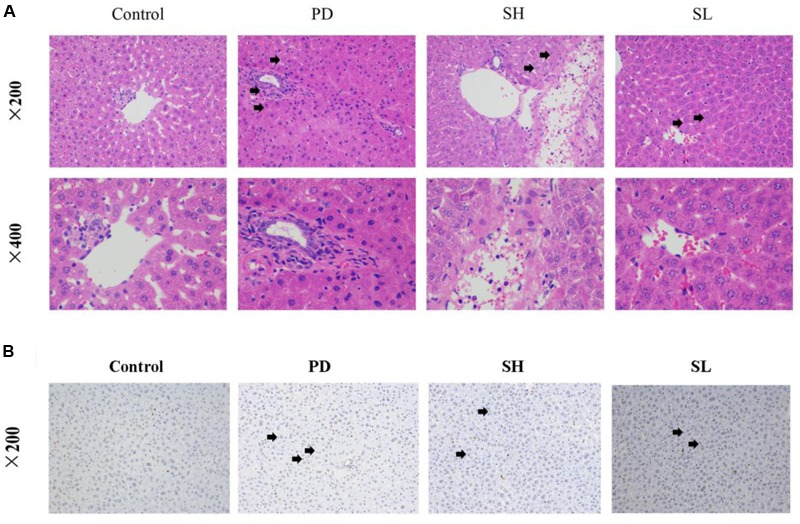
**The intervention effects of Sophocarpine on immunological liver injury.** Mice were treated with different doses of Sophocarpine. Positive expression in H&E and TUNEL Staining is displayed by the black arrows. **(A)** Representative photographs of 10 animals show the effect of Sophocarpine, which was confirmed by H&E. Blue is cytoplasm and red is nucleus; *N* = 10. **(B)** The effect of Sophocarpine pre-treatment on apoptotic cells; blue is nucleus and DAB showing positive expression is tan; *N* = 5. Poly I:C and D-GalN group (PD group), high-dose Sophocarpine group (SH group) and low-dose Sophocarpine group (SL group).

Evidence of the protective effect of Sophocarpine on Poly I: C/D-GalN-induced ILI was provided directly by histological examination. The histology of liver tissues revealed hemorrhagic lesions in the liver after it was exposed to Poly I: C/D-GalN, which were not observed in the control group. Specifically, the lesions exhibited centrilobular necrosis, immune cell infiltration into the portal tract and sinusoid hepatocyte ballooning, necrosis, and disintegration. Diffuse necrotic lesions with collections of inflammatory cells, especially in the perivenous region, which extends to the central zone, were observed after Poly I: C and D-GalN injection. More importantly, SH administration alleviated the severity of liver damage caused by Poly I:C/D-GalN, resulting in a situation similar to the control group. SL treatment moderately reduced both the inflammatory cell infiltration and histological damage (**Figure 2A**). Furthermore, pre-treatment with Sophocarpine alleviated signs of apoptosis (**Figure 2B**).

### Effect of Sophocarpine on Pro-inflammatory Cytokine Secretion

**FIGURE 3:**
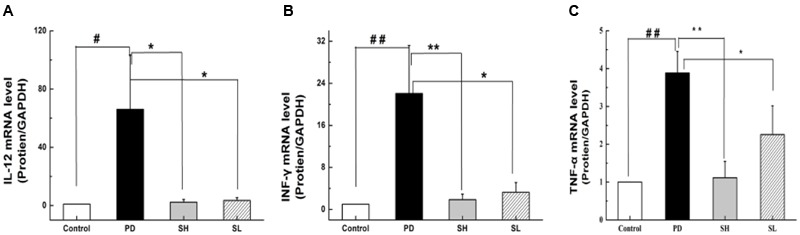
**Effect of Sophocarpine on pro-inflammatory cytokine secretion.** Mice were treated with different doses of Sophocarpine and detected by Q-PCR. **(A)** The mRNA levels of IL-12 and **(B)** INF-γ; **(C)** The concentration of TNF-α in the mice liver tissue; *N* = 10_._^#^*P* < 0.05, ^##^*P* < 0.01 compared with control group; ^∗^*P* < 0.05, ^∗∗^*P* < 0.01 compared with PD group. Poly I:C and D-GalN group (PD group), high-dose Sophocarpine group (SH group) and low-dose Sophocarpine group (SL group).

Playing a critical role in the innate immune system, NK cells are closely correlated with pro-inflammatory cytokine production. TNF-α, IL-12 and INF-γ are essential pro-inflammatory cytokines responsible for NK cell activity. Specifically, TNF-α can stimulate NK cells to secrete IL-12 and INF-γ. Furthermore, IL-12 can, in turn, activate NK cells, as suggested by an *in vivo* study (Hou et al., 2009). To examine the effect of Sophocarpine on the inflammatory response, the mRNA levels of TNF-α, IL-12 and INF-γ in the liver tissue were detected. Compared with the control group, the expression level of TNF-α in the PD group increased by approximately 3.8-fold. Moreover, the mRNA levels of INF-γ and IL-12 increased by nearly 22.1- and 66.1-fold, respectively. Sophocarpine significantly downregulated the expression of TNF-α by 71.2% in the SH group and by 41.9% in the SL group (**Figure 3C**). Furthermore, Sophocarpine decreased the mRNA levels of INF-γ and IL-12 by 91.6 and 96.7% in the SH group and by 68.1 and 94.7% in the SL group, respectively, compared with the PD group (**Figures 3A,B**).

### Sophocarpine Reduced NK Cell Accumulation in the Liver

**FIGURE 4:**
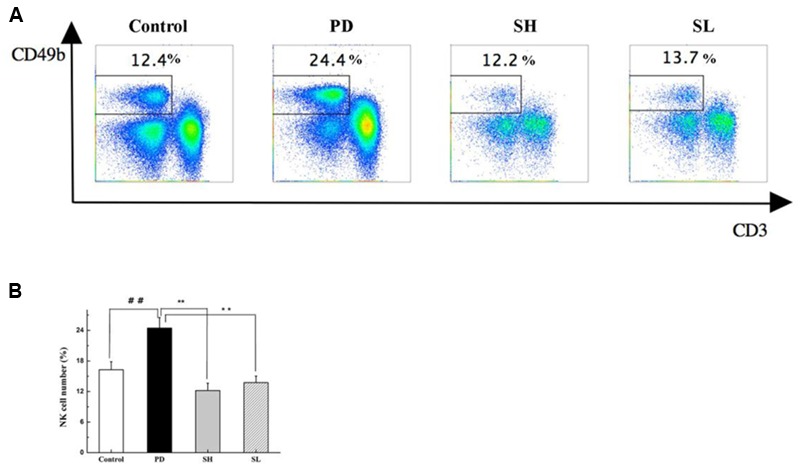
**The effect of Sophocarpine on the accumulation of NK cells in the liver.** Mice were treated with different doses of Sophocarpine for 12 days and injected with Poly I: C/D-GalN. Lymphocytes were isolated from the liver, and the surface expressions of NK cells, which were marked by CD3e and CD49b, were detected by flow cytometry. **(A,B)** Are the total number of NK cells in the liver calculated by multiplication with the respective percentage of lymphocytes; *N* = 10. ^##^*P* < 0.01 compared with control group; ^∗∗^*P* < 0.01 compared with PD group. Poly I: C and D-GalN group (PD group), high-dose Sophocarpine group (SH group) and low-dose Sophocarpine group (SL group).

Poly I:C injection preferentially induced NK cell recruitment to the liver (Dong et al., 2004). The effect of Sophocarpine on NK cell accumulation was analyzed using flow cytometry. As shown in **Figures 4A,B**, the proportion of NK cells in the liver increased by nearly twofold after Poly I: C/D-GalN injection, which was consistent with previous studies (Dong et al., 2004). After Sophocarpine treatment, the proportion of NK cells decreased by 50.1% in the SH group and by 43.8% in the SL group, thus exhibiting a dose-dependent relationship. These consequences suggested that Sophocarpine suppressed the accumulation of NK cells in the liver.

### Regulation of the NKG2D-DAP12 Signaling Pathway by Sophocarpine

**FIGURE 5:**
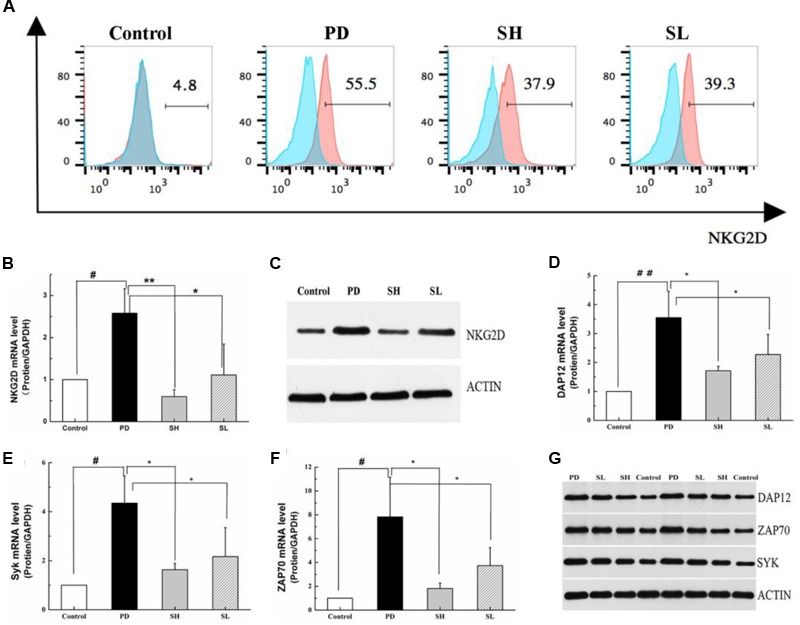
**Regulation of the NKG2D-DAP12 signaling pathway by Sophocarpine.** C57BL/6 mice were injected with Poly I:C and simultaneously intraperitoneally injected with D-GalN. Hepatic lymphocytes were isolated, and the expression of NKG2D on the NK cell surface was detected by flow cytometry **(A)**, whereas the expression of NKG2D **(B)**, DAP12 **(D)**, ZAP70 **(E)**, and Syk **(F)** were analyzed by Q-PCR; *N* = 10. Western blotting was used to analyze the protein level of NKG2D **(C)**, DAP12, ZAP70, and Syk **(G)**; *N* = 3. ^##^*P* < 0.01 compared with control group; ^∗∗^*P* < 0.05 compared with PD group. Poly I:C and D-GalN group (PD group), high-dose Sophocarpine group (SH group) and low-dose Sophocarpine group (SL group).

Natural killer cells express a repertoire of activating receptors, and NKG2D is reported to be a stimulatory immunoreceptor that exists on the NK cell membrane. Previous reports have demonstrated that human NK cells need to be ‘primed’ by stimulation initialized from NK cell surface receptors, which may trigger cytotoxicity in some abnormal conditions (Bryceson et al., 2006). Therefore, we measured the expression of NKG2D in mouse liver after Poly I: C/D-GalN induction due to its essential role in NK cell activation. As shown in **Figures 5A–C**, NKG2D expression was significantly higher in the PD group than that in the control group. Sophocarpine clearly decreased the expression level of NKG2D, and this effect was more pronounced in the SH group. NKG2D activation can induce changes both in its adaptor, DAP12, and in the down-stream signaling pathways, which may initialize NK cell-driven ILI. The association of NKG2D with DAP12 has significant consequences for signal transduction in that DAP12 possesses a canonical immunotyrosine-based activation motif (ITAM), which recruits Syk and ZAP70 tyrosine kinases (7) (Lanier et al., 1998). To clarify the mechanism underlying the inhibition of NK cell activation by Sophocarpine, we investigated the expression levels of DAP12, Syk, and ZAP70 (**Figures 5D–G**). Quantitative real-time PCR revealed that Poly I: C/D-GalN-induced ILI was associated with significantly upregulated DAP12, Syk, and ZAP70 mRNA levels, and these change could be reversed by Sophocarpine pre-treatment. Western blotting showed the expression of DAP12, Syk, and ZAP70 in liver tissue samples from pre-treated and untreated mice, and these assays demonstrated that Sophocarpine was capable of downregulating the expression of DAP12, Syk, and ZAP70. In addition, high-dose Sophocarpine was more effective than low-dose Sophocarpine (**Figures 5D–G**). Therefore, Sophocarpine might down-regulate the NKG2D-DAP12 signaling pathways to attenuate Poly I: C/D-GalN-induced ILI.

## Discussion

Due to an increasing interest in alternative medicine, many studies have attempted to elucidate the mechanism underlying the activity of the active compounds extracted from traditional Chinese herbs. In previous studies, Sophocarpine, a quinolizidine alkaloid extracted from *Radix Sophorae Subprostratae*, has been reported to exert activity on immune regulation (Zhang et al., 2007), and it is hepato-protective against hepatic fibrosis in rats (Qian, 2008). In our study, pre-treatment with Sophocarpine significantly and dose-dependently downregulated the levels of ALT and AST and upregulated the levels of TP, CHE and ALB. Furthermore, Sophocarpine pre-treatment significantly attenuated liver tissue damage, as evidenced by histology.

Natural killer cells, the major innate immune cells in the liver, play an important role in the innate immune response against liver injury (Kärre, 2002). Unlike T and B cells, NK cells lack variable receptors that recognize foreign antigens, but they express inhibitory and activating receptors to balance their activation (Samarakoon et al., 2009). The ability of various natural products or artificially synthesized compounds to recruit and activate NK cells has been studied (Wiltrout et al., 1989).

Polyinosinic-polycytidylic acid (Poly I: C) is a self-peptide that is used to induce chronic and severe pancreatitis in MRL/+mice (Jiang et al., 2005). Additionally, Poly I:C/D-GalN synergistically induced the production of INF-γ and TNF-α, although D-GalN alone did not elevate the levels of either cytokine, as previously reported (André et al., 2004; Rosen et al., 2004; Huang et al., 2013). The synergistic effect of Poly I: C/D-Ga1N on IL-12 is similar to that on INF-γ and TNF-α. In our studies, pre-treatment with Sophocarpine successfully inhibited the abnormal TNF-α, INF-γ, and IL-12 production induced by Poly I: C/D-GalN, which alleviated inflammation and led to liver damage. We also verified the therapeutic potential of Sophocarpine *in vivo* by demonstrating that this inhibitory effect is related to a decrease of accumulation of NK cells in the liver.

The balance between activating and inhibiting signals of NK cells is mediated by various receptors by which NK cell function is regulated (Martinović et al., 2015). NKG2D is the activating receptor on the surface of NK cells, and NK cell-mediated ILI by NKG2D-DAP12 (Lanier, 2005) the signaling pathway is a critical step. Interestingly and importantly, our study demonstrated that Sophocarpine could block the upregulation of both NKG2D and its adaptor, DAP12. Furthermore, Syk and ZAP70 tyrosine kinase, two key members in downstream signaling, were also downregulated following the administration of Sophocarpine, potentially indicating the mechanism underlying the therapeutic activity on ILI of Sophocarpine.

The expression of the NKG2D protein on the surface of NK cells requires interactions with its adapter protein to stabilize the receptor complex (Lanier, 2015). Mice express two isoforms of NKG2D protein as a result of alternative splicing. NK cells in resting mice express a longer (NKG2D-L) protein that is exclusively and non-covalently associating with the DAP10 adapter protein, whereas the activation of mice NK cells induces the alternative splicing of *Klrk 1*, resulting in a shorter (NKG2D-S) protein isoform that correlates with either DAP10 or DAP12 adapter protein (Diefenbach et al., 2002; Gilfillan et al., 2002). A previous study showed that association of NKG2D with DAP12 has significant consequences for signal transduction in that DAP12 possesses a canonicalITAM, which recruits and activates Syk and ZAP70 tyrosine kinases (Lanier et al., 1998), thus causing the activation of NK cells (Lanier et al., 1998; Cantoni et al., 1999; Wu et al., 2000; Jiang et al., 2002). Syk, a protein tyrosine kinase, plays a crucial role in intracellular signal transduction in hematopoietic cells and interacts with immune-receptor tyrosine-based activation motifs (ITAMs) located in the cytoplasmic domains of immune receptors. It couples the downstream signaling events of activated immunoreceptors that mediate diverse cellular responses, including proliferation, differentiation, and phagocytosis. The Syk family protein tyrosine kinase ZAP70 is expressed in T and NK cells and plays an essential role in regulating NK cell activation. DAP12 receptor complex is largely intact, whereas the transcription of cytokine genes is completely abrogated (Huntington et al., 2005; Hesslein et al., 2006; Mason et al., 2006). Thus, the two major functions of NK cells—cytokine secretion and cytotoxicity—are differentially regulated downstream of ITAM-containing NK receptors. As demonstrated in this study, the mRNA expression of Syk and ZAP70 was reduced by Sophocarpine. Moreover, the Western blot and immunohistochemistry results demonstrated that the protein levels of Syk and ZAP70 also declined.

This study demonstrated that Sophocarpine affects Poly I: C/D-GalN-induced ILI in mice. Sophocarpine significantly reduced the percentage of dead cells and ameliorated histopathology and morphological injury. Furthermore, it decreased the accumulation of NK cells by downregulating the NKG2D-DAP12 signaling pathway. Therefore, the present study suggests that Sophocarpine may be used to protect the liver from ILI. Although this application requires future clinical trials, our study still provides a promising therapeutic approach and meaningful reference for clinical practice.

## Materials and Methods

### Animal

All animal procedures were approved by the Animal Experiment Committee of the 302 Military Hospital of the People’s Liberation Army. Male C57BL/6 mice weighing 20 ± 2 g were obtained from the laboratory animal center of the Sibei Fu (Beijing) Laboratory Animal Science and Technology Corporation. Food and water were available ad libitum. All studies were performed in accordance with the guidelines of the Council on Animal Care of the Academia Sinica. The animals were randomly divided into four groups with twenty mice in each group. Sophocarpine (Us150218) was dissolved in normal saline and intragastrically administered to mice (60 mg/kg for the low dose and 120 mg/kg for the high dose) for 12 days. Finally, all animals in each group except the control group were intravenously injected with Polyinosinic-polycytidylic acid sodium (Poly I: C, Sigma) dissolved in pyrogen-fee saline at a concentration of 75 μg/kg (Huang et al., 2013). The mice were simultaneously intraperitoneally injected with D-galactosamine (D-GalN, Sigma) at a dose of 500 mg/kg (Huang et al., 2013). The mice were sacrificed 12 h after injection. The blood and liver were collected. The blood was centrifuged at 3000 *g* for 10 min to separate the serum without hemolysis. All of the serum and the rest of the liver tissue after hematoxylin-eosin staining were stored at -80°C.

### Blood Chemistry Analysis

The serum levels of AST, ALT, CHE, ALB, and TP were measured using an automatic biochemical analyzer (BS-300) and assay kits bought from Wan Tai Drd. All assays were performed according to the manufacturer’s protocol.

### Histopathological Evaluations and Immunohistochemical Observation

The liver tissues obtained from the experimental mice were fixed in 10% neutral buffered formalin, embedded in paraffin. The embedded tissues were cut into 5-μm-thick sections and disposed using hematoxylin-eosin (H&E) and TUNEL Staining. As for H&E staining, cytoplasm was stained in blue and the nucleus stained in red, while for TUNEL Staining the nucleus was stained in blue and DAB showing a positive expression was tan.

### Flow Cytometric Analysis of the Number of NK Cells and NK Cell-Membrane Receptors

The cells were stained with the indicated fluorescence-labeled mAba for surface antigens according to a standard protocol. For intracellular cytokine staining, the cells were fixed, permeabilized, and stained with APC-Cy7 Hamster Anti-Mice CD3e (BD Pharmingen 557596), FITC Rat Anti-Mouse CD49b (BD Pharmingen 553857), PE Rat anti-Mouse NKG2D (BD Pharmingen 558403). The stained cells were analyzed using a flow cytometer (BD FACSCanto^TM^ II), and the data were analyzed using FlowJo 7.6.1.

### RNA Extraction and Reverse-Transcriptase Quantitative PCR

The total RNA was extracted using TRIzol (Life Sciences) and reverse-transcribed in 50 μl of solution using the Revert kit (Thermo-Fisher). The mRNA level expression was quantitated using real-time PCR (Applied Biosystems 7500 Real Time PCR System) and a SYBR Green PCR Master Mix kit (life). The primers for NKG2D, DAP12, Syk, ZAP70, and GAPDH are given in **Table 1**. The gene expression values were calculated based on the ΔΔCt method as described previously using the mean of the respective cytokines in mock-treated mice as a calibrator. The relative quantities (RQs) were determined using the following equation: RQ = 2^(-ΔΔCt)^ (**Table 1**).

**Table 1:** Forward and reverse primers used in reverse transcription polymerase chain reaction.

Mouse gene	Sense primer (5′–3′)	Antisense primer (5′–3′)
IL-12	5′-TCA CAT CTG CTG CTC CAC AAG-3′	5′-AGC CAT GAG CAC GTG AAC C-3′
INF-γ	5′-TAC TGC CAC GGC AC A GTC AT-3′	5′-TGC CAG TTC CTC CAG ATA TCC-3′
NKG2D	5′-GGC TTG CCA TTT TCA AAG AG-3′	5′-TGA GCC ATA GAC AGC ACA GG-3′
GAPDH	5′-GTC CTC AGC GTA GCC CAA GAT G-3′	5′-CAA TGT GTC CGT CGT GGA TCT-3′
SYK	5′-CCA GAC AGA GGC CTT CAG AGA-3′	5′-AGC AGG CTC CTG TCC AGG TA-3′
ZAP70	5′-TGC GCA AGA AGC AGA TTG AC-3′	5′-ATG AGC CGC ACG ATG TAG G-3′
TNF-α	5′-GCC TCT TCT CAT TCC TGC TTG T-3′	5′-TTG AGA TCC ATG CCG TTG-3′
DAP12	5′-AGG CCC AGA GTG ACA CTT TCC-3′	5′-GTA CAC AGC CAG GGC AAT CAG-3′

### Western Blot for the Levels of NKG2D, DAP12, Syk, and ZAP70 in Liver Tissue

Mice liver tissue (0.15 g) was homogenized and subsequently lysed on ice with RIPA buffer (G2002) containing a protease inhibitor mixture. The sample was subjected to centrifugation at 12000 rpm and 4°C for 10 min to separate any debris. After centrifugation, 800 μl of supernatant was stored at -80°C. These samples were used to detect the presence of NKG2D, DAP12, ZAP70, and Syk. Antibodies against DAP12 (sc7853), NKG2D (sc-292424), Syk (D3Z1E), ZAP70 (A2195), and β-actin (GB13001-1) were diluted in a solution of 5% milk, Tris-buffered saline (TBS), and 0.05% Tween-20 to detect NKG2D, DAP12, Syk, ZAP70, and β-actin, respectively. After incubation with the appropriate peroxidase-conjugated secondary antibody, the membrane was washed in TBST for 5 min and detected using a Western blotting detection system (Quantity One, Bio-Rad Laboratories, USA).

### Study Design

**FIGURE 6:**
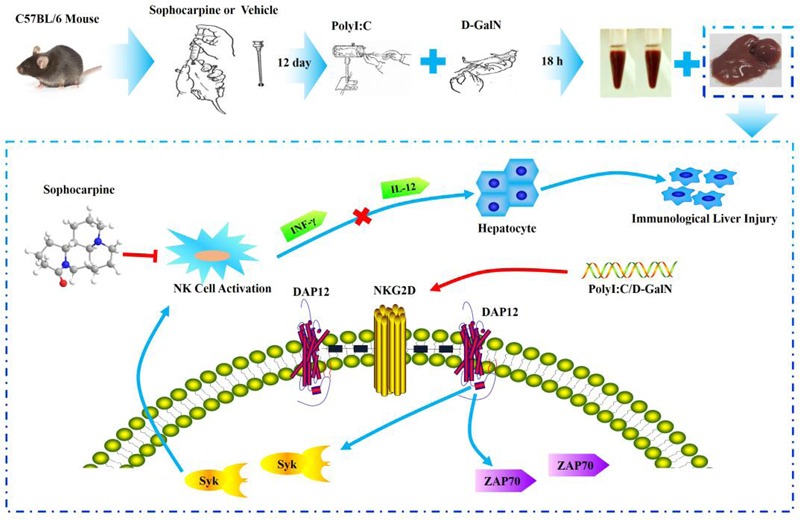
**Experimental flow chart and the probable protection mechanism of Sophocarpine in liver injury**.

The mice were randomly divided into four groups (control, PD, SH, and SL) in the whole study. Sophocarpine was administered intragastrically, and the control group and PD group were treated with saline for 12 days, once a day. After 12 days, all animals except those in the control group were intravenously injected with Polyinosinic-polycytidylic acid sodium (Poly I: C, Sigma) dissolved in pyrogen-fee saline at a concentration of 75 μg/kg (Huang et al., 2013) and simultaneously intraperitoneally injected with D-galactosamine (D-GalN, Sigma) at a dose of 500 mg/kg (Huang et al., 2013). D-GalN, when used as a sensitization agent, can increase the sensitivity of NK cells, and Poly I: C is able to activate NK cells. The mice were sacrificed 12 h after injection with the collection of blood and liver tissue for the hepatic index detection and NK cell related proteins (**Figure 6**).

### Statistics

The data are expressed as the mean ± standard deviation (SD) and were analyzed using the SPSS software (version 20.0; SPSS, Inc., Chicago, IL, USA). The differences between the group means were assessed based on the mean ± standard error of the mean (SEM). The differences were considered to be significant when *P* < 0.05 and highly significant when *P* < 0.01.

## Author Contributions

The authors Y-QH was responsible for primary data generation, analysis and writing the manuscript. Y-QH, Z-RY, J-BW, Y-LZ, and X-HX participated in the design of the study. P-YL, H-QZ, R-CY, Z-FB, and L-FW were involved the *in vivo* experimentation and technical work. J-YL and H-HL are responsible for the extensive statistical analyses.

## Conflict of Interest Statement

The authors declare that the research was conducted in the absence of any commercial or financial relationships that could be construed as a potential conflict of interest.

## Abbreviations

ILI: immunological liver injury
NK: natural killer
Poly IL: C and D-GalN group (PD group)
SL: low-dose Sophocarpine
SH: high-dose Sophocarpine
